# Editorial: Psychological intervention for suicidal ideation, behavior, and attempted suicide

**DOI:** 10.3389/fpsyt.2024.1497473

**Published:** 2024-10-14

**Authors:** Kazuki Matsumoto, Sayo Hamatani, Tushar Singh

**Affiliations:** ^1^ Division of Clinical Psychology, Kagoshima University Hospital, Research and Education Assembly Medical and Dental Sciences Area, Kagoshima University, Kagoshima, Japan; ^2^ Research Center for Child Mental Development, University of Fukui, Fukui, Japan; ^3^ Department of University, Banaras Hindu University, Varanasi, Uttar Pradesh, India

**Keywords:** pysychological intervetion, psychotherapy, self-harm behavior, suicidal behavior, suicidal ideation, suicide, suicide prevention

Can psychotherapy serve as an effective deterrent to the global suicide crisis, which claims approximately 720,000 lives annually? (1) With a multitude of factors, including mental illness, self-harm, and LGBTQ+ identity, contributing to heightened suicide risk (2). Psychological intervention holds promise as a preventive measure, offering early interventions based on suicide risk assessments and addressing individual mental health concerns (3). Unraveling the underlying psychological mechanisms of suicide is a critical first step toward developing novel solutions to this significant public health problem. This Research Topic presents the findings of 11 research groups that have explored psychological interventions or the risk factors targeting the process from suicidal ideation to suicide attempt (Oliveira et al., Ghadipasha et al., Reifels et al., Yu et al., Kılıçarslan et al., Valladares-Garrido et al., Werdin and Wyss, Chalker et al., Käll and Andersson, Beatty et al., Jobes and Rizvi).

A systematic review for suicide prevention in educational settings has highlighted both effective interventions and practical challenges, including Initiatives to suicide prevention, intervention, and post-suicide care (Oliveira et al.). A systematic review exploring the spatial and geographical factors associated with youth suicide identified specific groups and regions at higher risk, such as males, rural residents, and the unemployed (Ghadipasha et al.). Furthermore, a systematic review of suicide prevention measures in disaster and emergency contexts identified effective interventions while also highlighting the need for further research (Reifels et al.). In addition to these studies, this Research Topic presents the latest research findings from various perspectives, including the impact of physical exercise on suicide prevention (Yu et al.), the relationship between domestic violence and suicide (Kılıçarslan et al.), the link between romantic loss and suicidal ideation (Valladares-Garrido et al.), the challenges of suicide prevention in Europe (Werdin and Wyss), and future research directions (Chalker et al.).

The papers presented in this Research Topic offers a valuable resource to researchers, clinicians, and policymakers engaged in suicide prevention. It is our hope that the insights gained from this research will facilitate the development of enhanced support systems for those at risk of suicide, thereby contributing to solve suicidal problem.

## References

[1] World Health Organization. Suicide (2024). Available online at: https://www.who.int/news-room/fact-sheets/detail/suicide (Accessed 2-Sep-24).

[2] Mughal F Ougrin D Stephens L Vijayakumar L Kapur N. Assessment and management of self-harm and suicide risk in young people. BMJ. (2024) 386:e073515. doi: 10.1136/bmj-2022-073515 39103171

[3] Mann JJ Michel CA Auerbach RP. Improving suicide prevention through evidence-based strategies: A systematic review. Am J Psychiatry. (2021) 178:611–24. doi: 10.1176/appi.ajp.2020.20060864 PMC9092896 33596680

